# FAM3A enhances adipogenesis of 3T3-L1 preadipocytes via activation of ATP-P2 receptor-Akt signaling pathway

**DOI:** 10.18632/oncotarget.17578

**Published:** 2017-05-03

**Authors:** Yujing Chi, Jing Li, Na Li, Zhenzhen Chen, Liping Ma, Weikang Peng, Xiuying Pan, Mei Li, Weidong Yu, Xiangjun He, Bin Geng, Qinghua Cui, Yulan Liu, Jichun Yang

**Affiliations:** ^1^ Institute of Clinical Molecular Biology & Central Laboratory, Peking University People's Hospital, Beijing 100044, China; ^2^ Department of Gastroenterology, Peking University People's Hospital, Beijing 100044, China; ^3^ Department of Physiology and Pathophysiology, School of Basic Medical Sciences, Peking University Health Science Center, Key Laboratory of Cardiovascular Science of the Ministry of Education, Center for Non-coding RNA Medicine, Beijing 100191, China; ^4^ Department of Biomedical Informatics, School of Basic Medical Sciences, Peking University Health Science Center, Key Laboratory of Cardiovascular Science of the Ministry of Education, Center for Non-coding RNA Medicine, Beijing 100191, China

**Keywords:** FAM3A, PPARγ, adipogenesis, ATP, P2 receptor

## Abstract

FAM3A plays important roles in regulating hepatic glucose/lipid metabolism and the proliferation of VSMCs. This study determined the role and mechanism of FAM3A in the adipogenesis of 3T3-L1 preadipocytes. During the adipogenesis of 3T3-L1 preadipocytes, FAM3A expression was significantly increased. FAM3A overexpression enhanced 3T3-L1 preadipocyte adipogenesis with increased phosphorylated Akt (pAkt) level, whereas FAM3A silencing inhibited 3T3-L1 preadipocyte adipogenesis with reduced pAkt level. Moreover, FAM3A silencing reduced the expression and secretion of adipokines in 3T3-L1 cells. FAM3A protein is mainly located in mitochondrial fraction of 3T3-L1 cells and mouse adipose tissue. FAM3A overexpression increased, whereas FAM3A silencing decreased ATP production in 3T3-L1 preadipocytes. FAM3A-induced adipogenesis of 3T3-L1 preadipocytes was blunted by inhibitor of P2 receptor. In white adipose tissues of db/db and HFD-fed obese mice, FAM3A expression was reduced. One-month rosiglitazone administration upregulated FAM3A expression, and increased cellular ATP content and pAkt level in white adipose tissues of normal and obese mice. In conclusion, FAM3A enhances the adipogenesis of preadipocytes by activating ATP-P2 receptor-Akt pathway. Under obese condition, a decrease in FAM3A expression in adipose tissues plays important roles in the development of adipose dysfunction and type 2 diabetes.

## INTRODUCTION

Obesity, a clinical condition characterized by the excessive accumulation of body fat, is a strong risk factor for type 2 diabetes [1, 2]. PPARγ is one of the key transcription factors that regulate and promote adipogenesis, and its agonists such as rosiglitazone and pioglitazone exerted significant hypoglycemic effects [3]. However, one significant side effect of PPARγ agonists is bodyweight gain in both clinical studies and animal experiments [4, 5]. Transgenic mice with adipose-specific overexpression of PPARγ display obesity and increased global insulin sensitivity, and are protected against high fat diet (HFD)-induced insulin resistance and hyperglycemia [6]. In support, specific deletion of PPARγ in adipose causes lipodystrophy, global insulin resistance and hyperglycemia in mice [7]. This paradox of obesity and type 2 diabetes suggested that more studies are still needed to explore the role of adipose tissues in the development of type 2 diabetes under obese condition. Adipose dysfunction is also called adiposopathy or sick fat, which is mainly characterized by increased lipolysis and disturbed adipokine expression profile. Obese patients with adipose dysfunction are at higher risk of type 2 diabetes than those without adipose dysfunction [8–16]. When chronic exposure to excessive nutrients, expansion and adipogenesis of preadipocytes are necessary for storing lipid or glucose as TG [11, 14, 17]. Under the condition of excessive nutrients, an impairment in the capacity of preadipocyte adipogenesis will cause adipose dysfunction, leading to ectopic lipid deposition and disturbed adipokine expression/secretion [8, 18].

The family with sequence similarity 3 (FAM3) gene family is a cytokine-like gene family consists of four members designated FAM3A, FAM3B, FAM3C, and FAM3D, respectively. FAM3A is a target gene of PPARγ, and activation of PPARγ induced FAM3A expression in liver cells [19]. In the livers of obese diabetic mice and patients with non-alcoholic fatty liver disease (NAFLD), FAM3A expression was reduced. Hepatic activation of FAM3A markedly attenuated hyperglycemia, insulin resistance and fatty liver in obese diabetic mice via the activation of Akt signaling pathway [20]. So far, whether FAM3A regulates the adipogenesis of preadipocytes remains unknown.

In the current study, we reported that FAM3A enhances the adipogenesis of 3T3-L1 preadipocytes by activating ATP-P2 receptor-Akt signaling pathway. In white adipose tissues of obese diabetic mice, FAM3A expression was reduced. Rosiglitazone administration upregulated FAM3A expression in HFD-fed diabetic mouse tissues with improved adipokine profile and hyperglycemia. These findings suggested that a decrease in FAM3A expression in adipose tissues contributes to the development of adipose dysfunction and type 2 diabetes.

## RESULTS

### Rosiglitazone administration upregulated FAM3A expression in epididymal white adipose tissue of C57BL/6 mice

**Figure 1:**
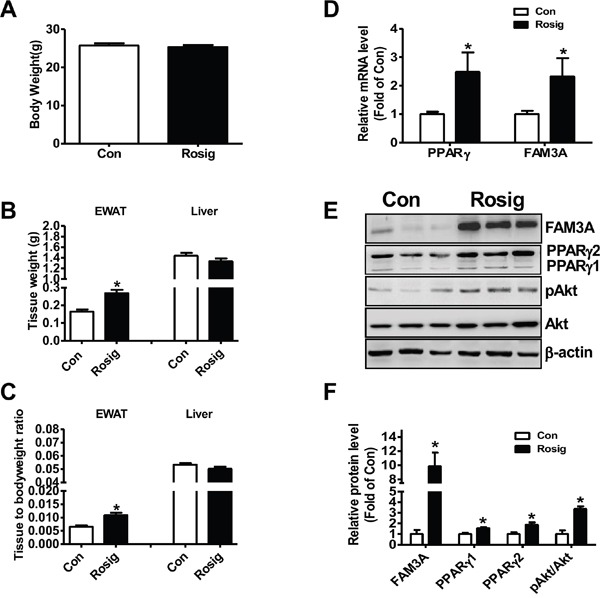
Rosiglitazone administration upregulated FAM3A expression in adipose tissues of C57BL/6 mice 8-10 week old C57BL/6 mice fed on normal diet were orally administrated with rosiglitazone for 1 month. **(A-C)** Rosiglitazone administration on bodyweight **(A)**, epididymal white adipose tissue (EWAT) and liver weight **(B)**, and tissue weight/bodyweight ratios **(C)**. **(D)** Rosiglitazone administration on the mRNA levels of PPARγ and FAM3A in adipose tissues. The primer for PPARγ mRNA analysis detected both PPARγ1 and PPARγ2 mRNA in adipose tissues in this study. **(E, F)** Rosiglitazone administration on the protein levels of PPARγ and FAM3A in adipose tissues. Representative gel images were shown in **(E)**, and quantitative data shown in **(F)**. Con: control mice treated with saline; Rosig: experiment mice treated with rosiglitazone. N=6-8 for protein assays, N=12-14 for mRNA assays, *p<0.05 versus control mice.

To initially determine whether FAM3A is involved in adipogenic process, C57BL/6 mice were orally administrated with rosiglitazone for one month, and then FAM3A expression in white adipose tissue was determined. Although rosiglitazone treatment failed to affect whole bodyweight (Figure 1A), it increased the weight of epididymal white adipose tissue (EWAT) (Figure 1B, 1C). The mRNA and protein levels of PPARγ and FAM3A were increased by rosiglitazone treatment in EWAT of mice (Figure 1D, 1E). Moreover, the phosphorylated Akt (pAkt) level was also increased by rosiglitazone treatment in EWAT of mice (Figure 1E, 1F).

### FAM3A expression was increased during the adipogenesis of 3T3-L1 preadipocytes

**Figure 2:**
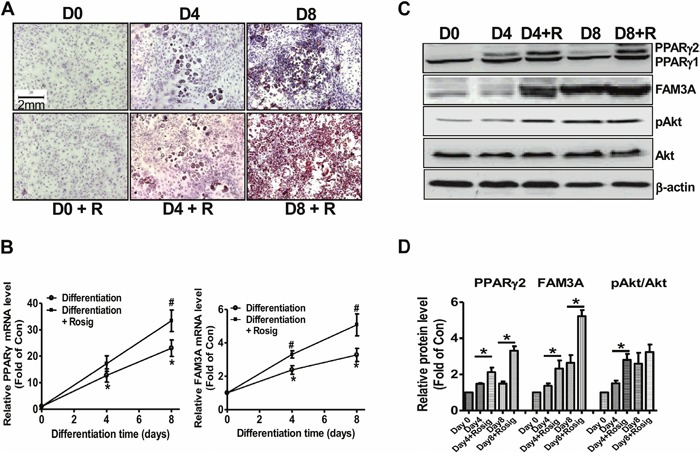
FAM3A expression was increased in differentiated 3T3-L1 preadipocytes **(A)** Representative images of differentiated 3T3-L1 cells with Oil Red O staining. The magnification power is 100. **(B)** The mRNA levels of PPARγ and FAM3A during differentiation of 3T3-L1 cells. The primer for PPARγ mRNA analysis detected both PPARγ1 and PPARγ2 mRNA in 3T3-L1 cells in this study. **(C, D)** The protein levels of PPARγ and FAM3A during differentiation of 3T3-L1 cells. Representative gel images were shown in **(C)**, and quantitative data shown in **(D)**. D0, D4, and D8 represented 0 day, 4 days and 8 days after induction of differentiation, respectively; R, in the presence of rosiglitazone. N=5-8, *p<0.05 versus D0 or control, #p<0.05 versus differentiation group without rosiglitazone at the corresponding time point.

To further confirm that FAM3A is involved in adipogenic process, its expression in differentiated 3T3-L1 preadipocytes was determined. When compared to the normally differentiated 3T3-L1 cells, Oil Red O staining revealed that rosiglitazone induced more neutral lipid deposition on differentiation Day 4 and Day 8 (Figure 2A). In normally differentiated 3T3-L1 cells, the mRNA levels of PPARγ and FAM3A were increased on Day 4 and Day 8 (Figure 2B, 2C) when compared with that on Day 0. Moreover, rosiglitazone induced further elevation in PPARγ and FAM3A mRNA levels when compared with normally differentiated cells (Figure 2B). The protein levels of PPARγ2 and FAM3A were increased (Figure 2C, 2D), whereas that of PPARγ1 remained unchanged during adipogenesis of 3T3-L1 cells (Figure 2C, 2D). pAkt level was increased on Day 4 and Day 8 when compared with Day 0. Rosiglitazone induced further elevation in PPARγ2 and FAM3A protein levels on Day 4 and Day 8, but only induced further increase in pAkt level on Day 4 but not Day 8 (Figure 2C, 2D). The mRNA levels of adipokines including aP2, CEBP/α, CEBP/δ, adiponectin and resistin were elevated during the adipogenesis of 3T3-L1 cells (Supplementary Figure 1). The mRNA levels of aP2, CEBP/α, adiponectin and resistin were further induced by rosiglitazone in 3T3-L1 cells (Supplementary Figure 1).

### FAM3A silencing inhibited the differentiation of 3T3-L1 cells

**Figure 3:**
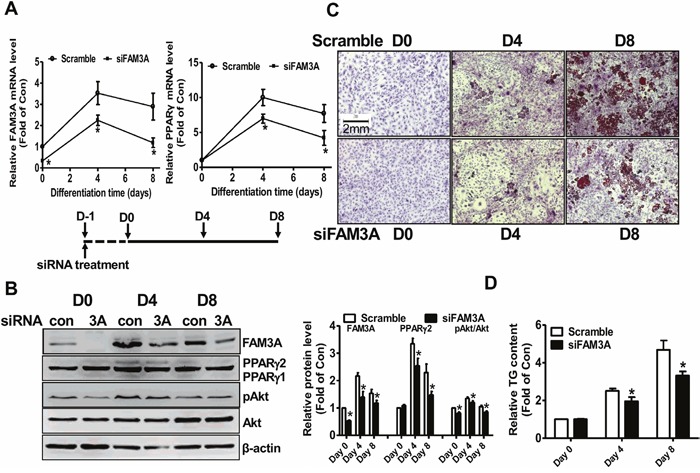
siRNA-mediated silencing of FAM3A inhibited the differentiation of 3T3-L1 preadipocytes **(A)** siFAM3A treatment on the mRNA levels of FAM3A and PPARγ in 3T3-L1 cells. **(B)** siFAM3A treatment on the protein levels of FAM3A and PPARγ in 3T3-L1 cells. Representative gel images were shown in left panel, and quantitative data shown in right panel. **(C)** Representative images of differentiated 3T3-L1 cells with Oil Red O staining after treatment with siFAM3A or scrambled siRNA. **(D)** Quantitative analysis of TG levels in differentiated 3T3-L1 cells. siFAM3A: cells treated with siFAM3A; scramble: cells treated with scrambled siRNA. N=6, *p<0.05 versus control groups treated with scrambled siRNA at the corresponding time point.

To directly evaluate the impact of FAM3A on the adipogenesis of 3T3-L1 preadipocytes, its expression was knockdown using siRNA interference. FAM3A mRNA level was reduced about 67% in 3T3-L1 preadipocytes after siFAM3A treatment for 24 hours, and remained lower on Day 4 and Day 8 when compared with control cells (Figure 3A). FAM3A knockdown reduced the mRNA level of PPARγ on Day 4 and Day 8 (Figure 3A). siFAM3A treatment significantly reduced the protein levels of FAM3A, PPARγ2 and pAkt on Day 0, Day 4 and Day 8 when compared with control cells. Oil Red O staining and quantitative assays revealed that FAM3A silencing significantly reduced neutral lipid deposition and adipogenesis of 3T3-L1 cells on Day 4 and Day 8 (Figure 3C, 3D). FAM3A silencing reduced the mRNA levels of adipokine aP2, C/EBPα, adiponectin and resistin, whereas it had little effect on that of C/EBPδ in 3T3-L1 cells on Day 8 (Supplementary Figure 2A-2E). FAM3A silencing reduced adiponectin secretion on Day 4, whereas it reduced resistin secretion on Day 4 and Day 8 in 3T3-L1 cells (Supplementary Figure 2F, 2G).

### FAM3A overexpression enhanced the differentiation of 3T3-L1 cells

**Figure 4:**
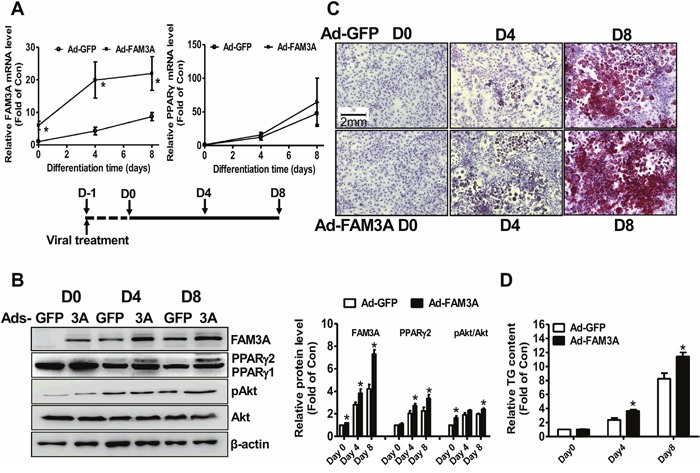
FAM3A overexpression enhanced the differentiation of 3T3-L1 preadipocytes **(A)** Ad-FAM3A treatment on the mRNA levels of FAM3A and PPARγ in 3T3-L1 cells. **(B)** Ad-FAM3A treatment on the protein levels of FAM3A and PPARγ in 3T3-L1 cells. Representative gel images were shown in left panel, and quantitative data shown in right panel. **(C)** Representative images of differentiated 3T3-L1 cells with Oil Red O staining after Ad-FAM3A or Ad-GFP treatment. **(D)** Quantitative analysis of TG levels in differentiated 3T3-L1 cells. N=5-8, *p<0.05 versus control groups treated with Ad-GFP at the corresponding time point.

FAM3A was overexpressed in 3T3-L1 cells to further confirm its adipogenic role in preadipocyte adipogenesis. On Day 0, Day 4 and Day 8 post viral infection, Ad-FAM3A-transduced cells exhibited significantly higher FAM3A mRNA level when compared with Ad-GFP-treated cells. FAM3A overexpression had no significant effect on the mRNA level of PPARγ (Figure 4A). Ad-FAM3A treatment significantly increased FAM3A protein level on Day 0, Day 4 and Day 8, and PPARγ2 protein level on Day 4 and Day 8 when compared with control cells. Moreover, FAM3A overexpression increased pAkt level on Day 0 and Day 8 (Figure 4B). Oil Red O staining and quantitative assays revealed that FAM3A overexpression enhanced neural lipid deposition and adipogenesis of in 3T3-L1 preadipocytes (Figure 4C, 4D). FAM3A overexpression failed to further increase the mRNA levels of adipokines (Supplementary Figure 3A-3E), and the secretion of adiponectin and resistin in 3T3-L1 preadipocytes (Supplementary Figure 3F, 3G).

### FAM3A enhanced the adipogenesis of 3T3-L1 preadipocytes via ATP-P2 receptor-Akt signaling pathway

**Figure 5:**
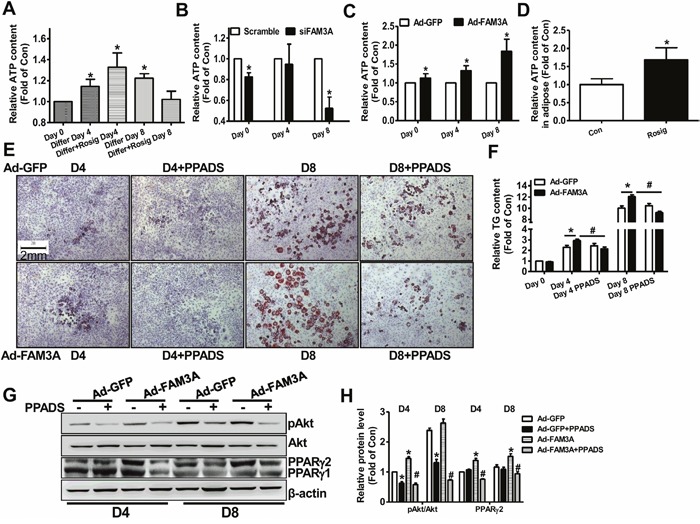
Inhibition of P2 receptor blocked FAM3A-induced adipogenesis of 3T3-L1 preadipocytes **(A-C)** Extracellular ATP content in differentiated 3T3-L1 cells **(A)**, siFAM3A-treated 3T3-L1 cells **(B)**, and Ad-FAM3A-treated 3T3-L1 cells **(C)**. ATP content in the medium of these cells were determined as described in experimental procedure. **(D)** ATP content in adipose tissues of C57BL/6 mice treated with rosiglitazone for 1 month. **(E)** Representative images of differentiated 3T3-L1 cells with Oil Red O staining after treatment with Ad-FAM3A or Ad-GFP in the absence or presence of PPADS. **(F)** Quantitative analysis of TG levels in differentiated 3T3-L1 cells in panel **(E)**. **(G, H)** Inhibition of P2 receptor blocked FAM3A-induced Akt activation in 3T3-L1 cells. Representative gel images were shown in **(G)**, and quantitative data shown in **(H)**. N=6, *p<0.05 versus control groups treated with Ad-GFP at the corresponding time point; #p<0.05 versus Ad-FAM3A-treated group.

Our previous study had revealed that FAM3A is a novel mitochondrial protein [20, 21]. FAM3A protein is also predominantly located in mitochondrial fraction of mouse adipose tissue and 3T3-L1 cells (Supplementary Figure 4A, 4B). In proliferated 3T3-L1 cells, extracellular ATP content was increased (Figure 5A). FAM3A silencing reduced extracellular ATP content on Day 0 and Day 8 (Figure 5B), whereas FAM3A overexpression elevated extracellular ATP content on all time points (Figure 5C). One-month rosiglitazone treatment upregulated FAM3A expression and increased cellular ATP content in adipose tissues of C57BL/6 mice (Figure 5D). To determine whether FAM3A enhances the adipogenesis of 3T3-L1 cells via ATP signaling pathway, P2 receptor (ATP receptor) signaling was blocked using PPADS. Because there is no significant adipogenesis on Day 0, the experiment of Day 0 had not been performed here. In Ad-GFP-treated cells, adipogenesis was not significantly affected by PPADS treatment on Day 4 and Day 8 (Figure 5E, 5F). In contrast, PPADS treatment significantly repressed FAM3A-stimulated adipogenesis on Day 4 and Day 8 (Figure 5E, 5F). In support, FAM3A-induced increase in pAkt and PPARγ2 protein levels was blunted by PPADS treatment (Figure 5G, 5H). Although PPADS treatment failed to affect PPARγ2 protein level and adipogenesis in Ad-GFP-infected cells, it reduced pAkt level on Day 4 and Day 8 (Figure 5G, 5H). On Day 4 and Day 8, PPADS treatment reduced PPARγ mRNA level in Ad-FAM3A-treated cells but not in Ad-GFP-treated cells (Supplementary Figure 5A). PPADS treatment reduced the mRNA levels of aP2 and resistin on Day 8 in Ad-FAM3A-treated cells (Supplementary Figure 5B-5F).

### FAM3A is reduced in adipose tissues of HFD mice and db/db mice

**Figure 6:**
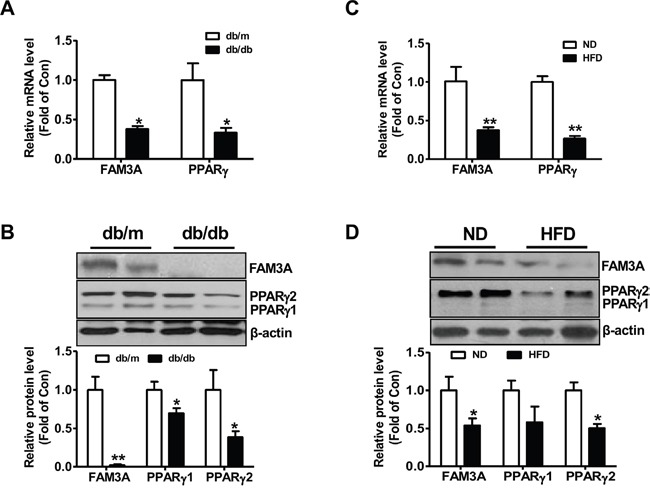
FAM3A expression was reduced in white adipose tissues of obese diabetic mice **(A, B)** In adipose tissues of db/db mice, the mRNA **(A)** and protein **(B)** levels of FAM3A and PPARγ were reduced when compared with db/m mice. **(C, D)** In adipose tissues of mice fed on HFD for 12 weeks, the mRNA **(C)** and protein **(D)** levels of FAM3A and PPARγ were reduced when compared with mice fed a normal chow. ND: normal diet; HFD: high fat diet. N=5-8, *p<0.05 versus control mice.

**Figure 7:**
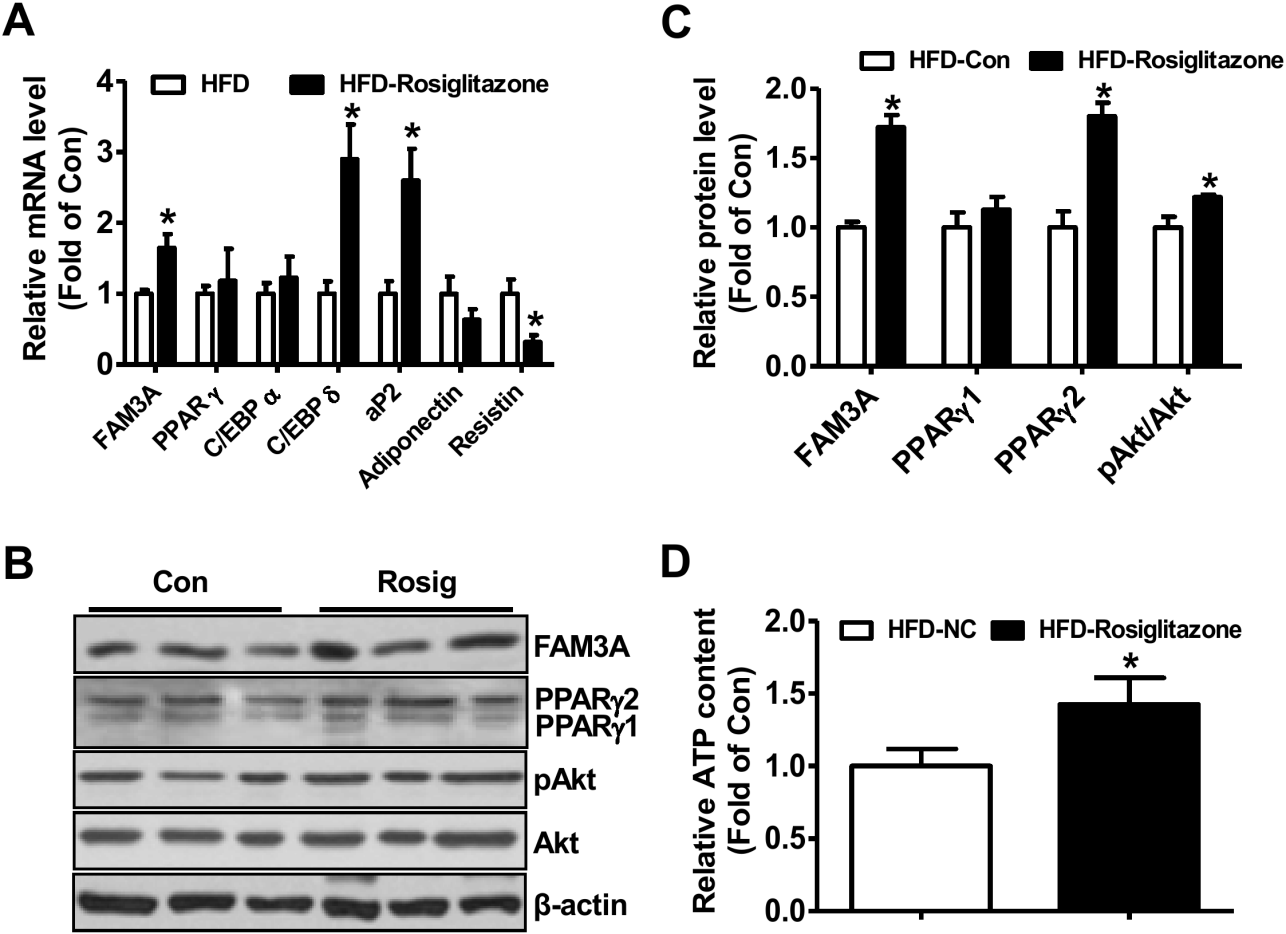
Rosiglitazone administration upregulated FAM3A expression in white adipose tissues of mice fed on HFD for 12 weeks C57BL/6 mice were fed on a HFD or normal chow for 12 weeks, followed by oral treatment with rosiglitazone for 1 month. **(A)** Rosiglitazone treatment on the mRNA levels of FAM3A and adipokines in adipose tissues. **(B, C)** Rosiglitazone treatment on the protein levels of FAM3A, PPARγ and pAkt in adipose tissues. **(D)** Rosiglitazone treatment increased cellular ATP content in adipose tissues. N=8, *p<0.05 versus control mice treated with saline.

The mRNA and protein levels of FAM3A and PPARγ were reduced in db/db (Figure 6A, 6B) and HFD mouse white adipose tissues (Figure 6C, 6D). PPARγ overexpression significantly activated the promoter activity of mouse FAM3A gene in 3T3-L1 cells, further supporting that PPARγ directly activated FAM3A expression in adipocytes (Supplementary Figure 6). Rosiglitazone treatment increased PPARγ2 protein level but not PPARγ1 protein level in adipose tissue of HFD-mice (Figure 7A, 7B). The mRNA level of FAM3A, and the protein levels of FAM3A and pAkt were increased in rosiglitazone-treated adipose tissue of HFD mice (Figure 7A-7C). The mRNA levels of aP2 and C/EBPδ were significantly increased, whereas that of resistin was decreased in rosiglitazone-treated adipose tissue (Figure 7A). The ATP content was also increased in HFD mouse adipose tissue by rosiglitazone treatment (Figure 7D).

**Figure 8:**
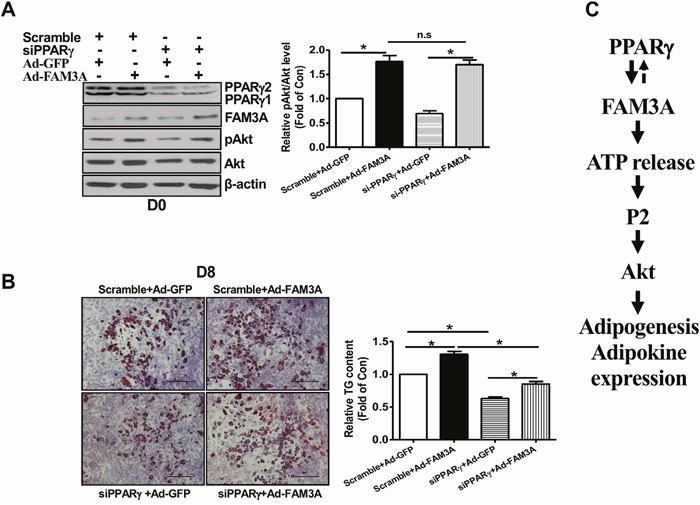
Knockdown of PPARγ failed to affect FAM3A-induced Akt activation in 3T3-L1 preadipocytes 3T3-L1 preadipocytes were transfected with siPPARγ (50 nM) or scrambled siRNA (50 nM) for 24 hours, and infected with Ad-GFP or Ad-FAM3A for 24 hours (Day 0). The infected cells were then induced for differentiation as above. **(A)** PPARγ silencing failed to affect FAM3A-induced Akt phosphorylation at Day 0. Representative gel images were shown in left panel, and quantitative data in right panel. N=4, *p<0.05 between indicated groups. **(B)** FAM3A promoted the adipogenesis of 3T3-L1 cells after PPARγ knockdown. Representative images of differentiated 3T3-L1 cells with Oil Red O staining were shown in left panel, and quantitative analyses of TG levels in right panel. N=3, *p<0.05 between indicated groups. **(C)** Schematic summary of the adipogenic effect of FAM3A in preadipocyte differentiation. FAM3A promotes the adipogenesis of preadipocytes by activating ATP-P2 receptor-Akt pathway.

siRNA-mediated knockdown of PPARγ failed to affect FAM3A-induced Akt phosphorylation at the beginning of 3T3-L1 preadipocyte differentiation (Figure 8A). Moreover, although PPARγ knockdown inhibited the differentiation of 3T3-L1 preadipocytes, FAM3A similarly promoted the differentiation of 3T3-L1 preadipocytes transfected with scrambled siRNA or siPPARγ (Figure 8B).

## DISCUSSION

So far, the biological functions of FAM3A remain largely unknown. FAM3A is a target gene of PPARγ [19]. In this study, gain- and loss-function evaluation revealed that FAM3A enhanced preadipocyte adipogenesis. FAM3A activates Akt via ATP-P2 receptor pathway in liver cells and vascular smooth muscular cells [20, 21]. There had been several lines of evidences suggesting that mitochondrial ATP production is associated with the adipogenesis of preadipocytes. Several key subunits of ATP synthase including ATP synthase alpha and beta subunits were increased during adipogenesis of 3T3-L1 cells [22]. Although addition of exogenous ATP alone failed to stimulate adipogenesis of 3T3-L1 preadipocytes, it augmented the adipogenic effects of other hormones by activating P2Y receptors [23]. Depletion of mitoferrin 1/2 reduced mitochondrial ATP production and repressed the adipogenesis of 3T3-L1 preadipocytes [24]. In support, overexpression of uncoupling protein 2 (UCP-2) impaired ATP production and induced the apoptosis of 3T3-L1 cells [25]. In adipose tissues of obese mice, several key genes controlling ATP production were reduced, but reversed by rosiglitazone treatment with the improvement of insulin resistance and hyperglycemia [26]. Activation of Akt pathways has been reported to play important roles in promoting adipogenesis of 3T3-L1 cells in several studies [27–30]. However, the impact of endogenous ATP production and its signaling pathway(s) on adipogenesis of preadipocytes still remains unclear.

Our findings demonstrated that FAM3A protein is predominantly located in mitochondrial fraction in adipose tissues and 3T3-L1 cells. FAM3A overexpression increased, whereas FAM3A silencing decreased ATP production in 3T3-L1 cells. Moreover, rosiglitazone administration also upregulated FAM3A expression, and increased ATP production in adipose tissues in C57BL/6 mice. Finally, PPADS, an antagonist of P2 receptor [20], blocked FAM3A-induced Akt activation and adipogenesis of 3T3-L1 cells. Clearly, FAM3A stimulates the adipogenesis of preadipocytes by activating ATP-P2 receptor-Akt signaling pathway. A decrease in FAM3A expression in adipose tissues might be a novel mechanism for explaining adipose dysfunction under obese condition [8, 18]. Restoration of FAM3A expression in adipose tissue may represent potential therapeutical strategy for correcting adipose dysfunction. In Ad-GFP-infected 3T3-L1 cells, PPADS treatment also reduced Akt phosphorylation but failed to significantly affect adipogenesis. This suggested that ATP-P2 receptor-Akt signaling axis is not the only pathway triggering adipogenesis in basal condition without FAM3A overexpression. In contrast, PPADS repressed FAM3A-induced Akt activation and adipogenesis in 3T3-L1 cells, indicating that ATP-P2 receptor-Akt pathway plays a decisive role in FAM3A-induced preadipocyte adipogenesis. In support, it had been previously reported that addition of exogenous ATP alone fails to stimulate adipogenesis, but it augments the adipogenic effects of dexamethasone and 3-isobutyl-1-methylxanthine in 3T3-L1 preadipocytes [23].

Regarding the role of FAM3A regulating adipogenic process, several issues should also be noted. FAM3A silencing inhibited the adipogenesis and adipokine expression/secretion in 3T3-L1 preadipocytes. However, although FAM3A overexpression enhanced the adipogenesis of 3T3-L1 preadipocytes, it failed to further increase adipokine expression/secretion. It is possible that adipokine expression are already at high levels during the adipogenesis, thus FAM3A overexpression fails to further elevate their levels in 3T3-L1 cells. Mice with adipose-specific deletion of PPARγ exhibit lipodystrophy and dramatically decreased serum adiponectin and resistin levels when compared with wild type mice [7]. In contrast, although mice with adipose-specific overexpression of PPARγ exhibit increased adipose mass, the serum adiponectin and resistin levels remained unchanged when compared with wild type mice [6]. Although FAM3A is a target gene of PPARγ, overexpression or silencing of FAM3A increases or decreases PPARγ2 protein level during adipogenesis. It had been reported that Akt activation upregulated PPARγ expression and activity by inactivating FOXO1 or activating mTOR during the adipogenesis of 3T3-L1 cells [31–34]. Our result further indicated that FAM3A-induced Akt activation is not dependent on PPARγ at the beginning of 3T3-L1 differentiation. So, FAM3A-mediated increase in PPARγ2 expression is likely due to long term Akt activation. It is also possible that an increase in PPARγ2 protein level is the consequence of 3T3-L1 preadipocyte maturation in case of FAM3A overexpression. Overall, there is a cross-talk between FAM3A and PPARγ2 expression during preadipocyte differentiation. It had been previously reported that in adipose tissue of fasting mice, PPARγ2 protein was reduced, whereas PPARγ1 protein remained unchanged when compared with that of fed mice [35]. Mice with PPARγ2 deletion exhibit reduced white adipose tissues when compared with wild type mice [36]. These findings suggested that PPARγ2 plays a crucial role in the regulation of preadipocyte adipogenesis. It should also be noted that FAM3A expression may also be regulated by other factors such as nutrients, other transcription factors or miRNAs beyond PPARγ during preadipocytes adipogenesis. Further study are still needed to clarify this crosstalk and its precise role in preadipocyte adipogenesis using mice with specific deletion of FAM3A and/or PPARγ in white adipose tissue in the future.

In summary, FAM3A plays important roles in regulating preadipocyte adipogenesis via the enhancement of ATP production, which activates Akt proliferative pathway(s) through P2 receptors. Under obese condition, a decrease in FAM3A expression in adipose tissue could contribute to the development of adipose dysfunction and type 2 diabetes (Figure 8C).

## MATERIALS AND METHODS

### Experimental animals

Eight-week-old male C57BL/6 mice and 8 to 12-week-old male db/db and db/m mice on a C57BSK background (Jackson Laboratory, USA) were used in this study. db/db and db/m mice were age matched. C57BL/6 mice were fed with a 45% high fat diet (HFD) or normal diet (ND) for 12 weeks [20]. Normal or HFD-fed C57BL/6 mice were daily administrated with rosiglitazone (AVANDIA, 30 mg/kg/day) for 30 days (Control mice were administrated with saline). Adipose tissues in this study are referred to be the epididymal white adipose tissues. All procedures involving experimental animals were approved by the Institutional Animal Care and Use Committee of Peking University Health Science Center that complies with the Guide for the Care and Use of Laboratory Animals published by the US National Institutes of Health (NIH Publication No. 85–23, revised 1996).

### Cell culture and differentiation

Mouse 3T3-L1 preadipocytes were cultured in DMEM supplemented with 10% FBS and penicillin-streptomycin at 37°C in 5% CO_2_. Two days post confluence (designed as Day 0), the cells were stimulated with MDI (0.5mM IBMX, 1μM dexamethasone and 10μg/mL insulin) induction media. In rosiglitazone group, 10μM rosiglitazone (Sigma) was added with MDI in Day 0. Two days after MDI (Day 2) change the media to Insulin Media (10μg/mL insulin). Two days later (Day 4) change media to 10% FBS/DMEM. Feed cells with 10% FBS/DMEM every two days. 3T3-L1 cells were collected on Day 0, Day 4 and Day 8 for further analyses, respectively.

### Overexpression or knockdown of FAM3A in 3T3-L1 preadipocytes

3T3-L1 preadipocytes were infected with 50 MOI of Ad-GFP or Ad-FAM3A for 24 hours before induction of differentiation (Day -1). PPADS (50μM) was added to media from Day 0 to Day 8 every 24 hours. 3T3-L1 cells were collected on Day 0, Day 4 and Day 8 for further analyses, respectively. To knockdown FAM3A gene expression, 3T3-L1 preadipocytes were transfected with 50 nm siFAM3A mixture against FAM3A cDNA coding sequence synthesized by Beijing Biolino Co., Ltd. (siRNA sequences were listed in Supplementary Table 1. Scrambled siRNA sequences from the same company were used as control) for 24 hours before induction of differentiation (Day -1). Cells were collected on Day 0, Day 4 and Day 8 for analysis, respectively.

### Oil Red O staining

At Day 0, Day 4 and Day 8 after induction, 3T3-L1 cells were fixed with 4% formaldehyde for 30min and then subjected to Oil Red O staining for 1 h to visualize neutral lipid deposition in the cells. Relative intracellular TG content was quantified by eluting Oil Red O staining with 100% isopropyl alcohol and measured at 570 nm with a microplate reader.

### Quantitative PCR assays

Total RNA of cells and tissues were isolated using TRIzol reagent (Invitrogen). The complementary DNA was synthesized using RevertAid First Strand cDNA Synthesis Kit (Thermo). Quantitative PCR was performed with the Biorad System using SRBR Green 1 (TOYOBO) as a fluorescent probe. Target gene mRNA level was normalized to that of β-actin in the same sample as described previously using 2^−ΔΔCt^ methods [19, 20]. All of the primer sequences used in this study are listed in Supplementary Table 2.

### Western blot analyses

Adipose tissues and cells were lysed in Roth lysis buffer. 60μg cell protein and 80μg adipose tissue protein were separated by 12% SDS-PAGE, and then transferred to the NC (nitrocellulose filter) membrane. The membrane was incubated in 1:500-1:1000 primary antibody. Primary antibodies were: anti-PPARγ antibodies (Santa Cruz Biotechnology, sc-7196), anti-FAM3A antibodies (Sigma), anti-pAkt (Ser473) and anti-Akt antibodies (Cell Signaling Technology). After incubated with the second antibody, the membrane was exposed to ECL. After immunoblotting assays, the membrane was stripped with 0.2N NaOH and re-probed for β-actin as loading controls.

### ELISA

Cell culture medium of 3T3-L1 cells on differentiation Day 0, Day 4 and Day 8 were collected and centrifuged at 12000 rpm at 4°C for 5 minutes, and then the supernatant were maintained for determination of adipokine levels. The levels of adiponectin and resistin were determined using ELISA Kit (Cloud-Clone Corp. SEA605Mu and SEA847Mu) according to the manufacturer's instruction.

### Mitochondria isolation

Mitochondria were isolated from adipose tissue or 3T3-L1 cells using the Mitochondria/Cytosol Fractionation Kit (Pierce). In brief, 3T3-L1 cells or adipose tissue were homogenized in Mito-Cyto extraction buffer, and then the lysate was centrifuged twice at 800g for 5 minutes to pellet the nucleus and cell debris. The supernatant was collected and centrifuged at 10,000 g for 10 min to pellet mitochondria, and then the mitochondria were washed at least three times with lysis buffer to avoid cytoplasmic proteins contamination.

### ATP content determination

ATP content in the cells and medium was determined using ATP-Lite Assay Kit (Vigorous Biotechnology Beijing Co., Ltd) as detailed previously [20]. For determination of relative ATP level in the cells or tissues, the ATP content values (nmol) were first normalized to the protein content (nmol/mg.protein) in the same sample, and then normalized to the control values. For determination of relative ATP level in the medium, the absolute concentration was determined and normalized to the control value.

### siRNA knockdown of PPARγ in 3T3-L1 preadipocytes

3T3-L1 preadipocytes were transfected with siRNA against both PPARγ1 and PPARγ2 (50 nM) or scrambled siRNA (50 nM) mixture using VigoFect (Vigorous Biotechnology, Beijing, China) for 24 hours, and then infected with Ad-GFP or Ad-FAM3A for 24 hours (Day 0). The infected cells were further induced for differentiation as above. The siRNA sequences were provided in Supplementary Table 1.

### Statistical analysis

Data are presented as mean±SEM. Statistical significance of differences between groups was analyzed by unpaired t test. Statistical significance was set at P<0.05.

## SUPPLEMENTARY FIGURES AND TABLES



## Abbreviations

aP2: adipocyte fatty acid-binding protein
C/EBPα: CCAAT-enhancer-binding protein α
C/EBPδ: CCAAT-enhancer-binding protein δ
EWAT: epididymal white adipose tissue
FAM3A: family with sequence similarity 3 member A
HFD: high fat diet
IBMX: 3-isobutyl-1- methylxanthine
MOI: multiplicity of infection
NAFLD: non-alcoholic fatty liver disease
PANDER: pancreatic derived factor
PI3K: phosphatidylinositol 3 kinase
PPARγ: peroxisome proliferator-activated receptor γ
siRNA: small interference RNA
TG: triglycerides
UCP-2: uncoupling protein 2
VSMCs: vascular smooth muscular cells
