# Supplementary material for: FAM3A enhances adipogenesis of 3T3-L1 preadipocytes via activation of ATP-P2 receptor-Akt signaling pathway
Source: Oncotarget. 2017 May 3;8(28):45862–73. doi: 10.18632/oncotarget.17578 (PMC5542233; doi:10.18632/oncotarget.17578)
Supplement: Supplementary file 1 [file oncotarget-08-45862-s001.pdf]

FAM3A enhances adipogenesis of 3T3-L1 preadipocytes via activation of ATP-P2 receptor-Akt signaling pathway

SUPPLEMENTARY MATERIALS

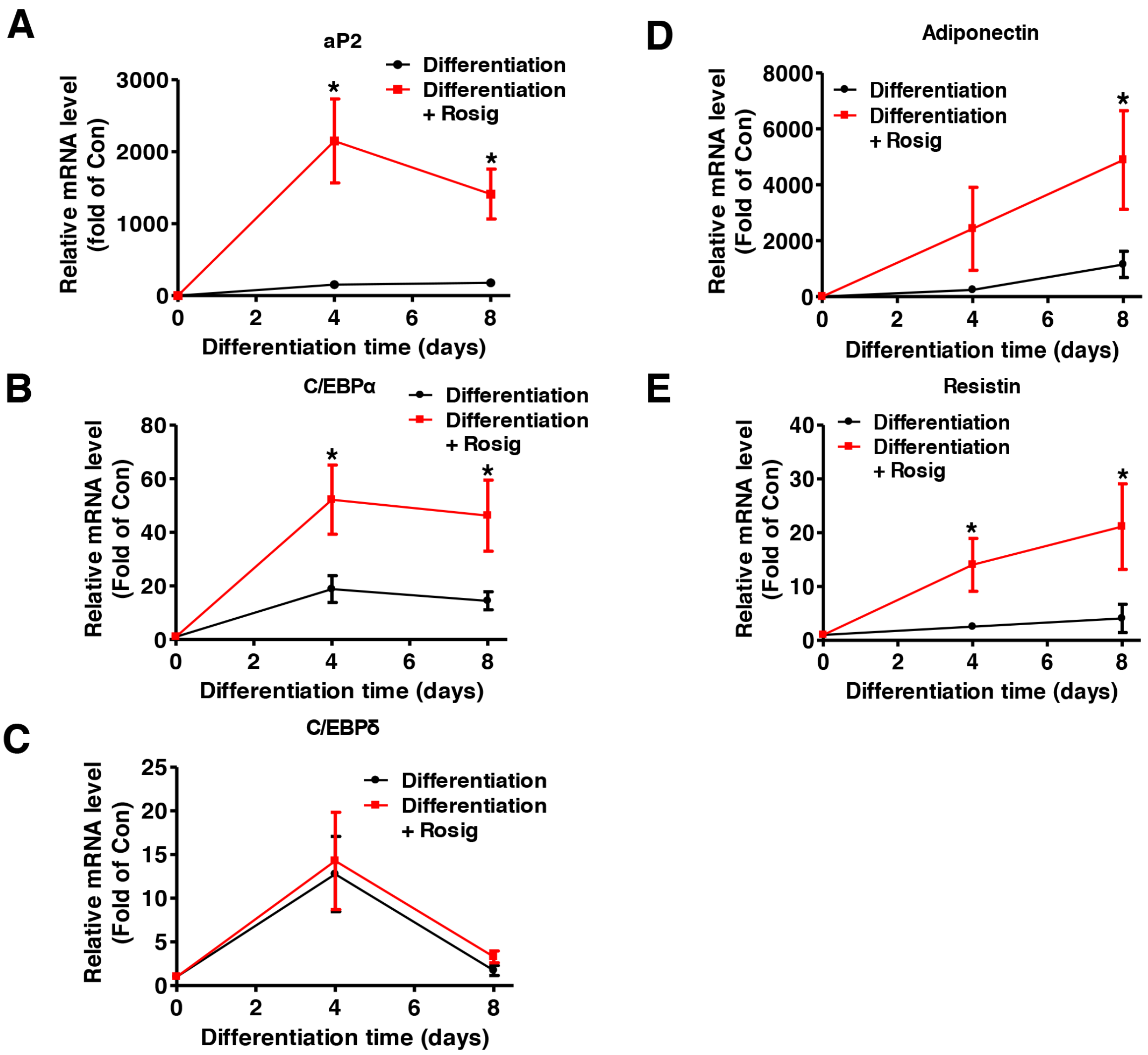

Supplementary Figure 1: Change in the mRNA levels of adipokines in differentiated 3T3-L1 cells in the absence or presence of rosiglitazone. **(A-E)** The mRNA levels of aP2 **(A)**, C/EBPα **(B)**, C/EBPδ **(C)**, adiponectin **(D)** and resistin **(E)** during adipogenesis. N=6, *p<0.05 versus normal differentiation group without rosiglitazone treatment at the corresponding time point.

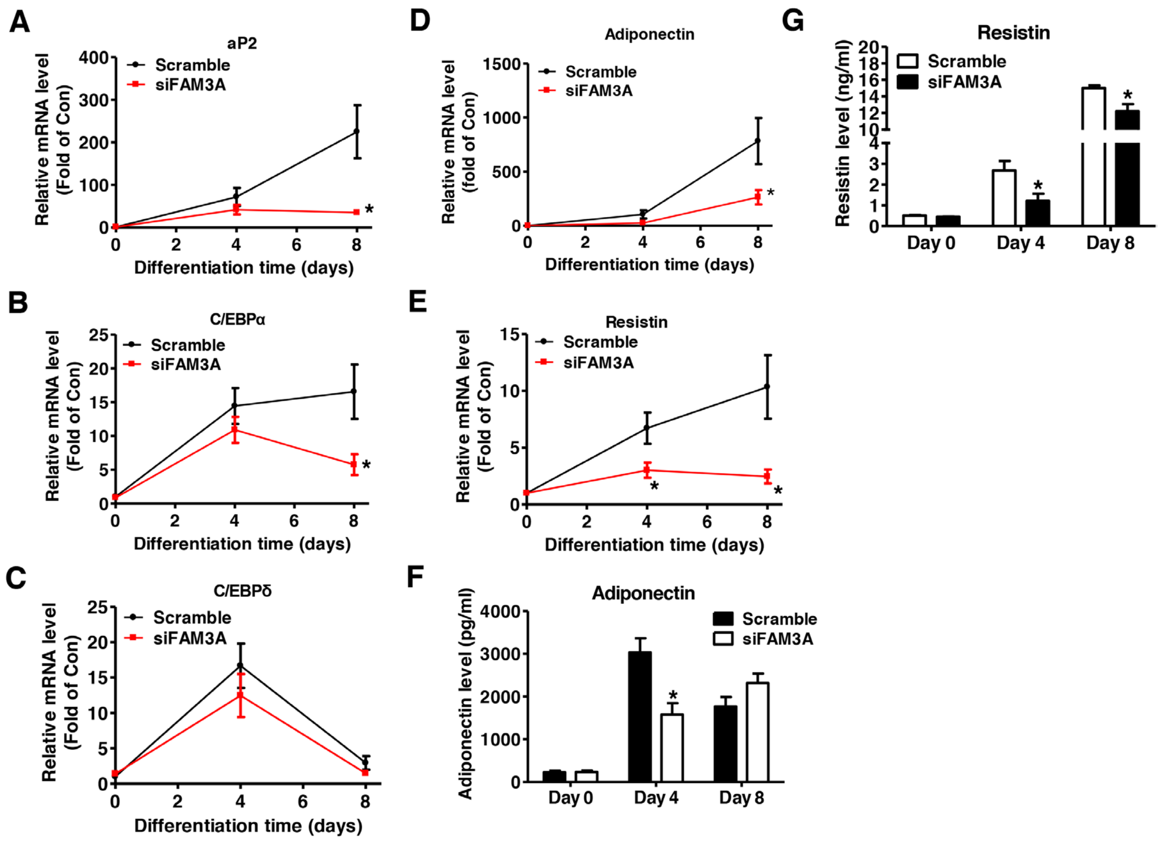

Supplementary Figure 2: FAM3A silencing on expression and secretion of adipokines of differentiated 3T3-L1 cells. **(A-E)** The mRNA levels of aP2 **(A)**, C/EBPα **(B)**, C/EBPδ **(C)**, adiponectin **(D)** and resistin **(E)** during adipogenesis. **(F, G)** FAM3A silencing on adipokine secretion in differentiated 3T3-L1 cells. Adiponectin **(F)** and resistin **(G)** protein levels in the medium supernatant were determined using ELISA. N=6, *p<0.05 versus group treated with scrambled siRNA at the corresponding time point.

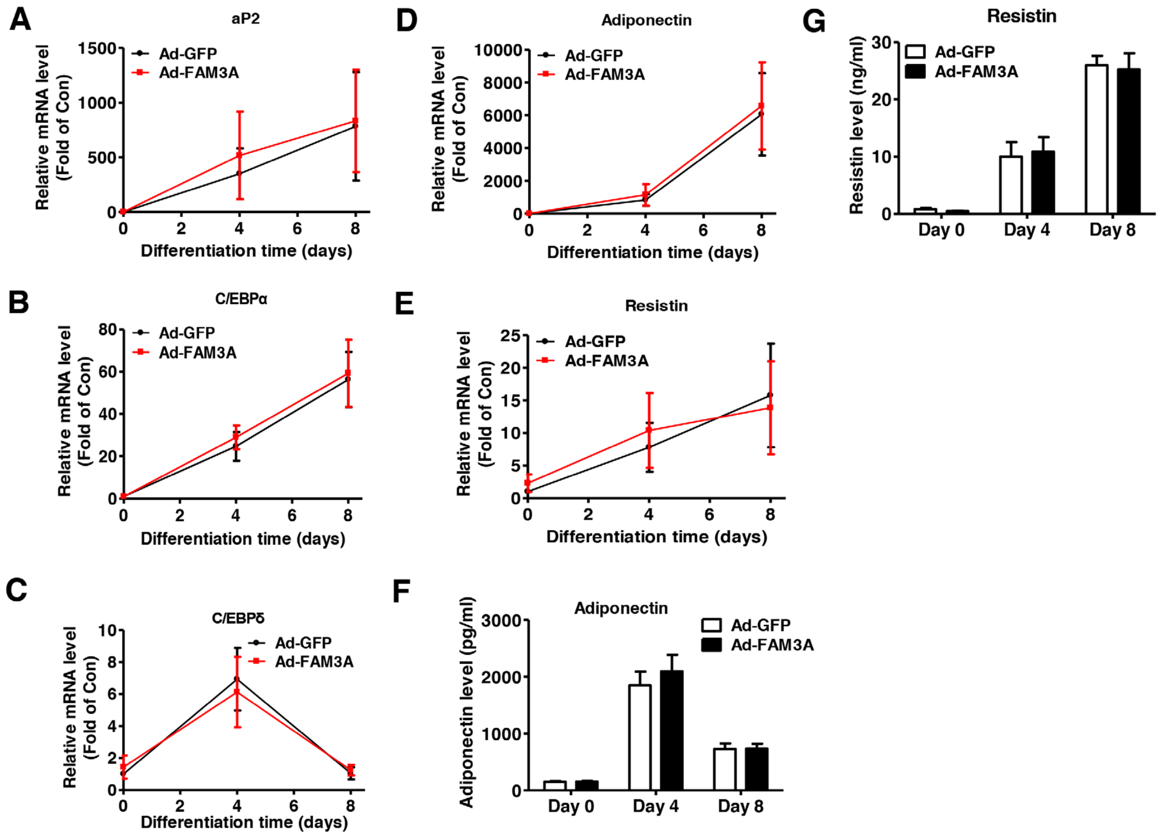

Supplementary Figure 3: FAM3A overexpression on expression and secretion of adipokines of differentiated 3T3-L1 cells. **(A-E)** The mRNA levels of aP2 **(A)**, C/EBPα **(B)**, C/EBPδ **(C)**, adiponectin **(D)** and resistin **(E)** during adipogenesis. **(F, G)** FAM3A overexpression on adipokine secretion in differentiated 3T3-L1 cells. Adiponectin **(F)** and resistin **(G)** protein levels in the medium were determined using ELISA. N=6, three is no significant difference between two groups at each corresponding time point.

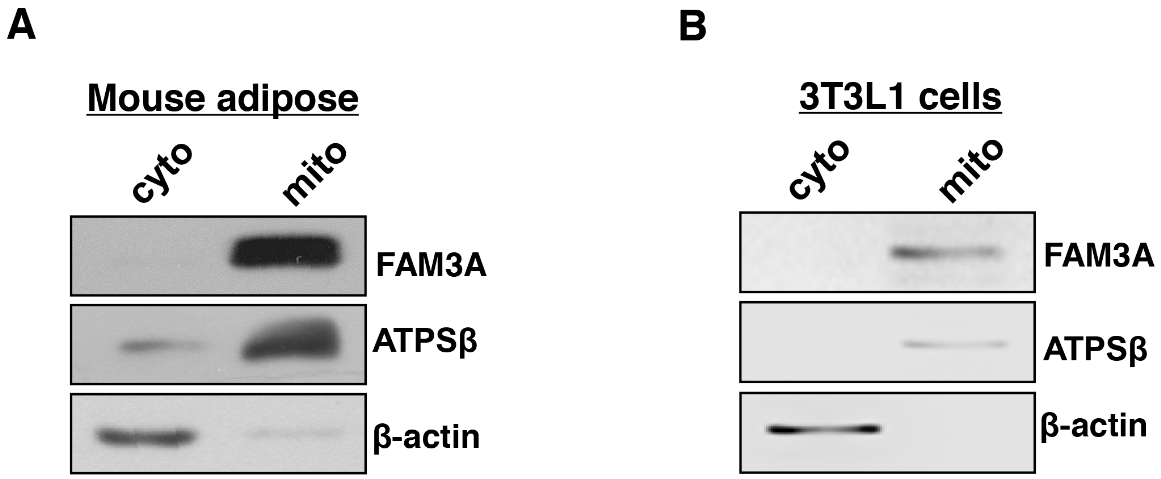

Supplementary Figure 4: FAM3A protein is mainly located in mitochondrial fraction of adipocytes. Mitochondria were isolated as described in the experimental procedure. **(A)** FAM3A protein is located in mitochondria of mouse adipose tissues. **(B)** FAM3A protein is located in mitochondria of 3T3-L1 preadipocytes. The gel images were the representatives of three independent experiments. Cyto: cytosolic fraction; mito, mitochondrial fraction; ATPSβ: ATP synthase β subunit, mitochondrial marker.

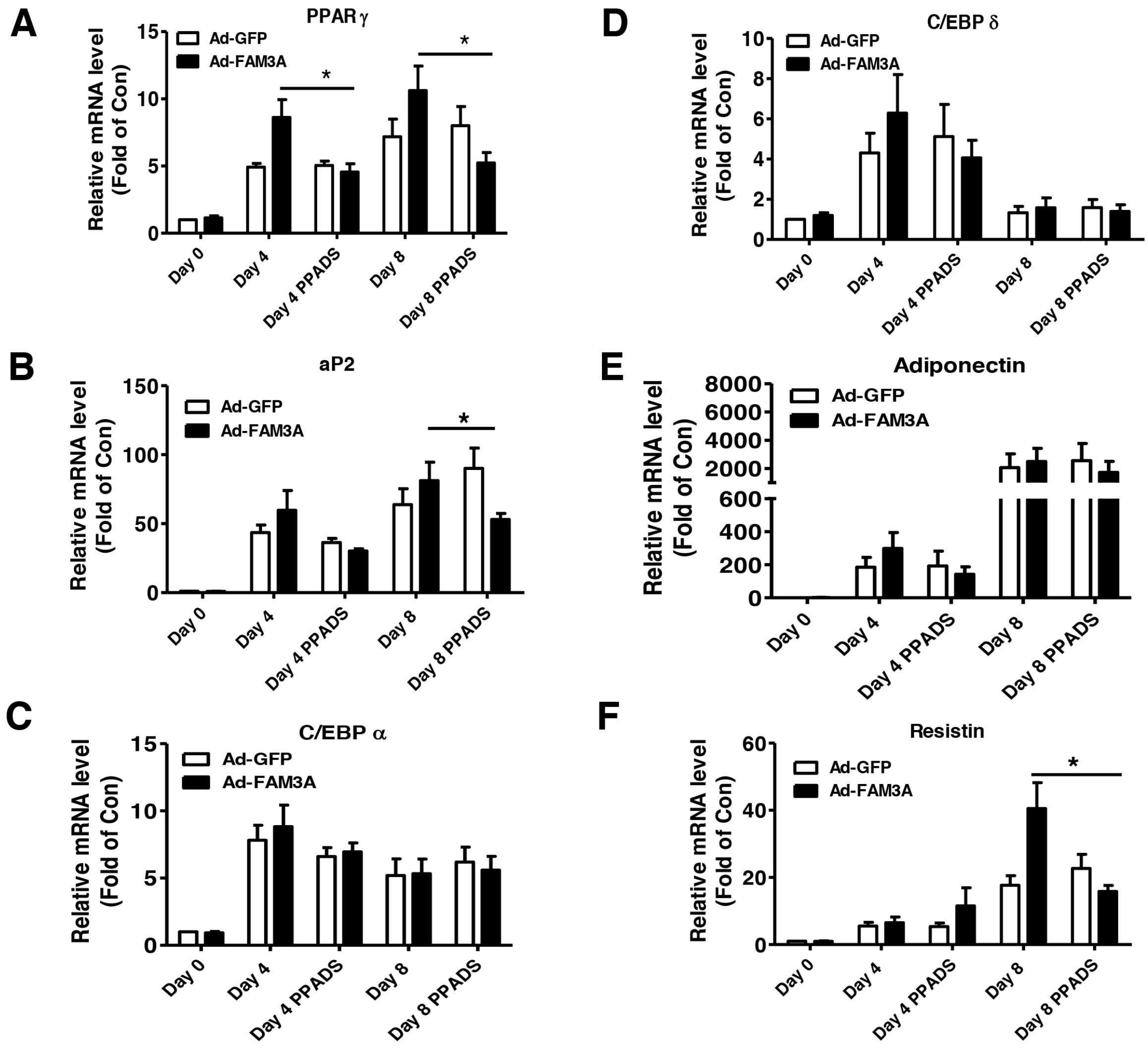

Supplementary Figure 5: Inhibition of P2 receptor on the mRNA levels of PPARγ and adipokines in differentiated 3T3-L1 cells. **(A)** The mRNA levels of PPARγ. **(B-F)** The mRNA levels of aP2 **(B)**, C/EBPα **(C)**, C/EBPδ **(D)**, adiponectin **(E)** and resistin **(F)** during adipogenesis. N=6, *p<0.05 between two indicated groups.

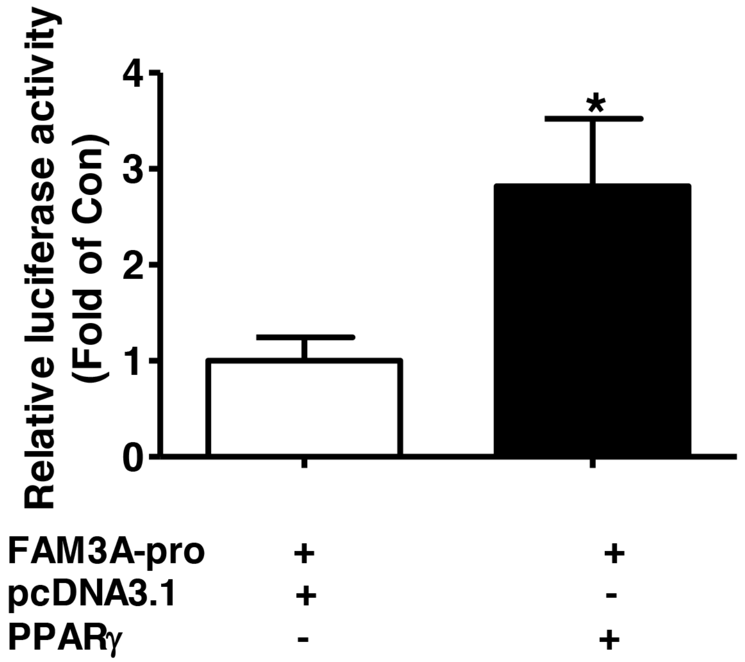

Supplementary Figure 6: PPARγ overexpression activated the reporter activity of mouse FAM3A gene promoter. The description of mouse FAM3A gene promoter reporter and the method for luciferase activity assay were detailed in previous study [19]. N=4, *$p<0.05$ versus control.

Supplementary Table 1: siRNA sequences against of mouse FAM3A and PPARγ genes

Dulex name	Sense seq	Antisense seq
FAM3A-1(M)	5'-CCUCGAAUUCAGCAGUUCUdTdT-3'	5'-AGAACUGCUGAAUUCGAGGdTdT-3'
FAM3A-2(M)	5'-GCACCUGAGCUUUCGAAUAdTdT-3'	5'-UAUUCGAAAGCUCAGGUGCdTdT-3'
FAM3A-3(M)	5'-CCAAAGGUGUGCAGAACAAdTdT-3'	5'-UUGUUCUGCACACCUUUGGdTdT-3'
PPARγ-1(M)	5'-GCCUAUGAGCACUUCACAATT-3'	5'-UUGUGAAGUGCUCAUAGGCTT-3'
PPARγ-2(M)	5'-GCAUUUCUGCUCCACACUATT-3'	5'-UAGUGUGGAGCAGAAAUGCTT-3'
PPARγ-3(M)	5'-GCAUCUCCACCUUAUUAUUTT-3'	5'-AAUAAUAAGGUGGAGAUGCTT-3'
PPARγ-4(M)	5'-CCAUCCGAUUGAAGCUUAUTT-3'	5'-AUAAGCUUCAAUCGGAUGGTT-3'

M: mouse origin.

Supplementary Table 2: List of oligonucleotide primer pairs used in real time PCR

Target gene	Sense primer	Antisense primer
FAM3A	5'-TCATGAGCAGCGTCAAAGAC-3'	5'-AGGGTACCTTCATGCAGTGG-3'
PPARγ1/2	5'-GACCAGCTGAACCCAGAGTC-3'	5'-GATGGCCACCTCTTTGCTCT-3'
aP-2	5'-GCGTGGAATTCGATGAAATCA-3'	5'-CCCGCCATCTAGGGTTATGA-3'
C/EBP α	5'-GGTGCGTCTAAGATGAGGGA-3'	5'-CCCCCTACTCGGTAGGAAAA-3'
C/EBP δ	5'-CTGAACGACCTATACCTCAGACC-3'	5'-AGCTTCTCTCGCAGTCCAGT-3'
Adiponectin	5'-TGTTGGAATGACAGGAGCTG-3'	5'-CGAATGGGTACATTGGGAAC-3'
Resistin	5'-TGCTGAATGTCCATCCATGTG-3'	5'-GGATCCTCACACAGGGAGTTG-3'
β-actin	5'-AGCCATGTACGTAGCCATCC-3'	5'-GCTGTGGTGGTGAAGCTGTA-3'

Note: The origin of primers referred to mouse.
